# Deciphering the Enigma: Lignocaine Resistance After Scorpion Envenomation

**DOI:** 10.7759/cureus.81128

**Published:** 2025-03-25

**Authors:** Karthiga Madheswaran, C V Srinivedha, Rinku George, Hemanth Kumar V R, Karthikeyan Padmanabhan

**Affiliations:** 1 Department of Oral and Maxillofacial Surgery, Indira Gandhi Institute of Dental Sciences, Sri Balaji Vidyapeeth, Puducherry, IND; 2 Department of Dentistry, Sri Balaji Vidyapeeth, Puducherry, IND; 3 Department of Anesthesiology and Critical Care, Mahatma Gandhi Medical College and Research Institute, Puducherry, IND; 4 Department of Otolaryngology - Head and Neck Surgery, Mahatma Gandhi Medical College and Research Institute, Puducherry, IND

**Keywords:** fibrotomy, lignocaine resistance, oral submucous fibrosis, ropivacaine, scorpion envenomation

## Abstract

Lignocaine, an amide-type local anesthetic (LA), is routinely used in dentistry. Resistance to lignocaine is rare, and it can occur due to various reasons, such as genetic mutations or variations in the local environment where it is injected. One reason might be scorpion envenomation.

We report a case of a 39-year-old female patient with a chief complaint of a gradual reduction in mouth opening for the past five years, with a history of scorpion bite. The patient had a scorpion bite 18 years back. She started developing symptoms of oral submucous fibrosis five years ago. The patient exhibited resistance to lignocaine, after which alternate agents were tried. We found that ropivacaine was effective as a LA, enabling airway management via cricothyrotomy and subsequent nasal intubation, following which bilateral fibrotomy, bilateral coronoidectomy, and bilateral nasolabial flap reconstruction were performed.

The resistance to lignocaine is due to modifications in the sodium channels. Scorpion venom affects the local environment surrounding the nerve and has systemic effects as well. As an alternative to lignocaine, bupivacaine and ropivacaine can be tested for sensitivity.

In patients with a recent history of scorpion bites, there is a higher chance of resistance to lignocaine. Therefore, evaluating the patients with a history of scorpion bites for resistance to lignocaine before any procedure is mandatory.

## Introduction

Local anesthesia is vital in dentistry, potentially easing pain and discomfort for patients undergoing extractions, third molar removal, and root canal treatments. These local anesthetic (LA) agents act by binding to sodium channels within cells, thus inhibiting the influx of sodium and preventing cell depolarization, which leads to the disruption of the transmission of action potential [1]. This mechanism helps obstruct pain signals from reaching the brain, thus resulting in less discomfort to patients undergoing extractions.

A commonly used LA in dentistry is lignocaine, which comes under the amide group. Failure of local anesthesia occurs due to various reasons, including technical errors, inadequate medication efficacy, or the presence of a local infection [2]. Unusual responses to LAs occur from mutations in sodium channels since they hinder nerve conduction by targeting these sodium channels.

Several factors, including sodium channel issues, genetic variations, and other physiologic conditions, can cause resistance to LAs, although that is fairly infrequent. Some of the conditions that might contribute to this resistance are Ehlers-Danlos syndrome (EDS), complex regional pain syndrome, and Crohn’s disease [3,4]. Moreover, the history of scorpion bites has been recognized to alter the LA action. Due to the high antigenic property of the venom of scorpions, there is a probability to induce the formation of antibodies against LAs, possibly causing genetic mutations in receptors that result in resistance to these agents [5].

Only a few case reports have been documented about the resistance to LAs because of scorpion bites. This present case describes a patient with a history of scorpion bites and lignocaine resistance diagnosed with oral submucous fibrosis and Grade IV trismus, necessitating surgical intervention. Any patient with a history of scorpion bites should be evaluated properly since, in this case, the scorpion bite happened 18 years back, and the patient is still resistant to lignocaine.

## Case presentation

A 39-year-old female presented to our outpatient department with a chief complaint of restricted mouth opening for the past five years. The patient was asymptomatic five years back but subsequently had difficulty in mouth opening, which progressed gradually to the current state of nil mouth opening. The patient also had stiffness in the bilateral buccal mucosa. The patient also reported a 13-year history of betel nut and tobacco use at a frequency of six to seven times daily, as well as a remote history of a scorpion sting that took place 18 years ago. The type of scorpion is unknown. Since the scorpion bite occurred 18 years back, the patient does not possess the medical records about the treatment given.

**Figure 1:**
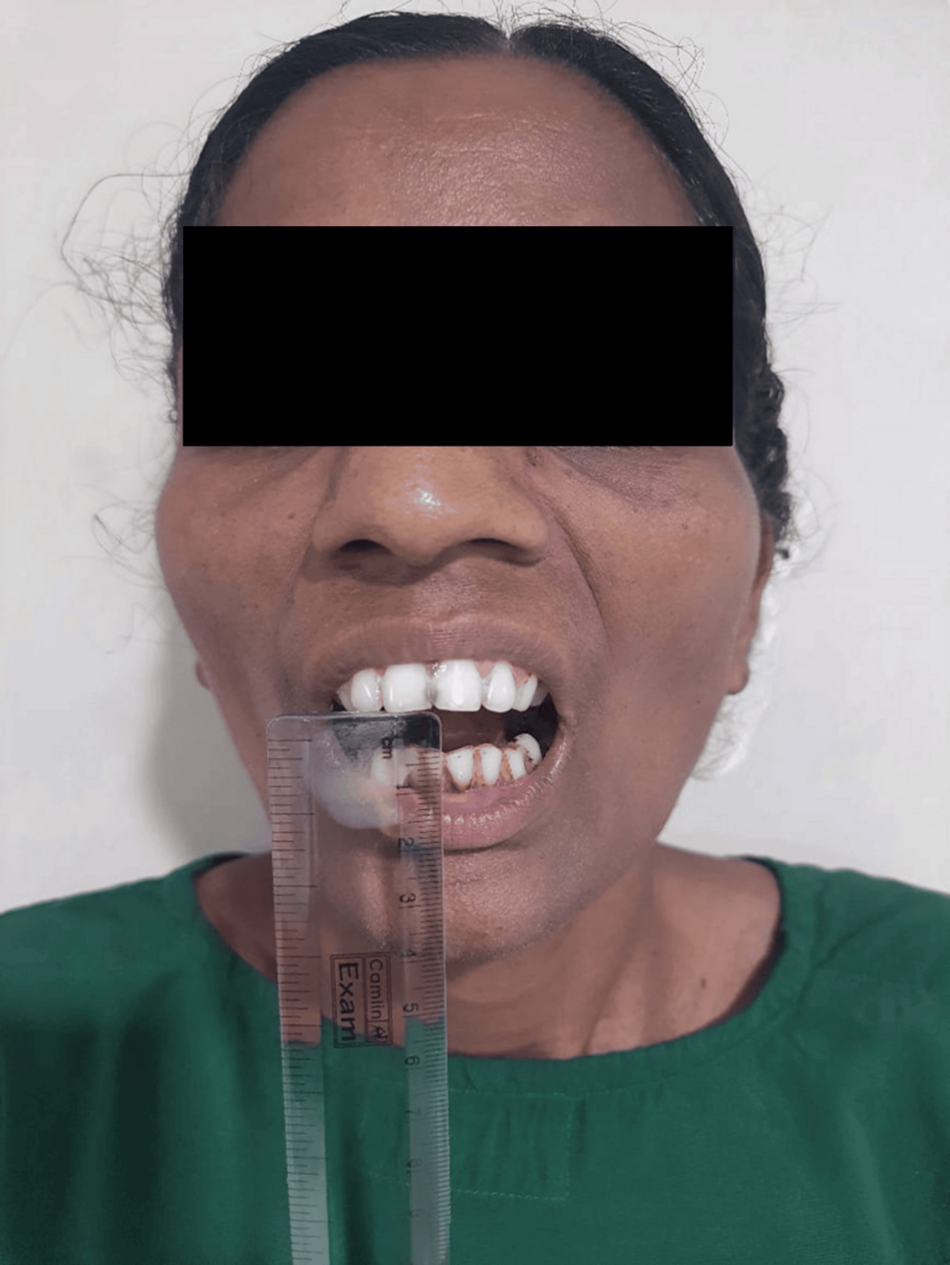
Preoperative mouth opening less than 5 mm

Clinical examination revealed a maximum mouth opening of approximately 5 mm without the presence of facial asymmetry (Figure 1). A palpable temporomandibular joint was noted, alongside restrictions in bilateral lateral jaw movements. Palpation revealed the existence of fibrous bands extending bilaterally in the buccal mucosa, coupled with tenderness and blanching of the labial mucosa. Based on these clinical findings, the patient was diagnosed with oral submucous fibrosis, classified as Stage S4 (clinical) and Stage M4 (functional) according to the criteria established by More et al. [6].

A preanesthetic evaluation was conducted, and the patient was scheduled for the procedure under general anesthesia. As a standard practice at our institution, a test dose of lignocaine was administered the day before surgery. Nasal packing with a 2% lignocaine solution was also performed to assess its effectiveness; however, the LA effect was insignificant even after a few minutes. Then, an intradermal patch test was done and found to be insensitive.

Given the patient's restricted mouth opening, intubation presented considerable challenges. Due to the lack of facilities for fiberoptic intubation in the institution, we had to consider alternate ways to safely intubate the patient. Consequently, the plan was to establish a patent airway using needle cricothyrotomy or tracheostomy under local anesthesia, subsequently converting to general anesthesia. Due to the patient’s insensitivity to lignocaine, alternative LAs, specifically ropivacaine, were explored. A test dose of ropivacaine was administered, and following a lack of adverse reactions, it was injected over a large area of the forearm to evaluate sensitivity. The patient demonstrated a positive response to the LA effect of ropivacaine.

**Figure 2:**
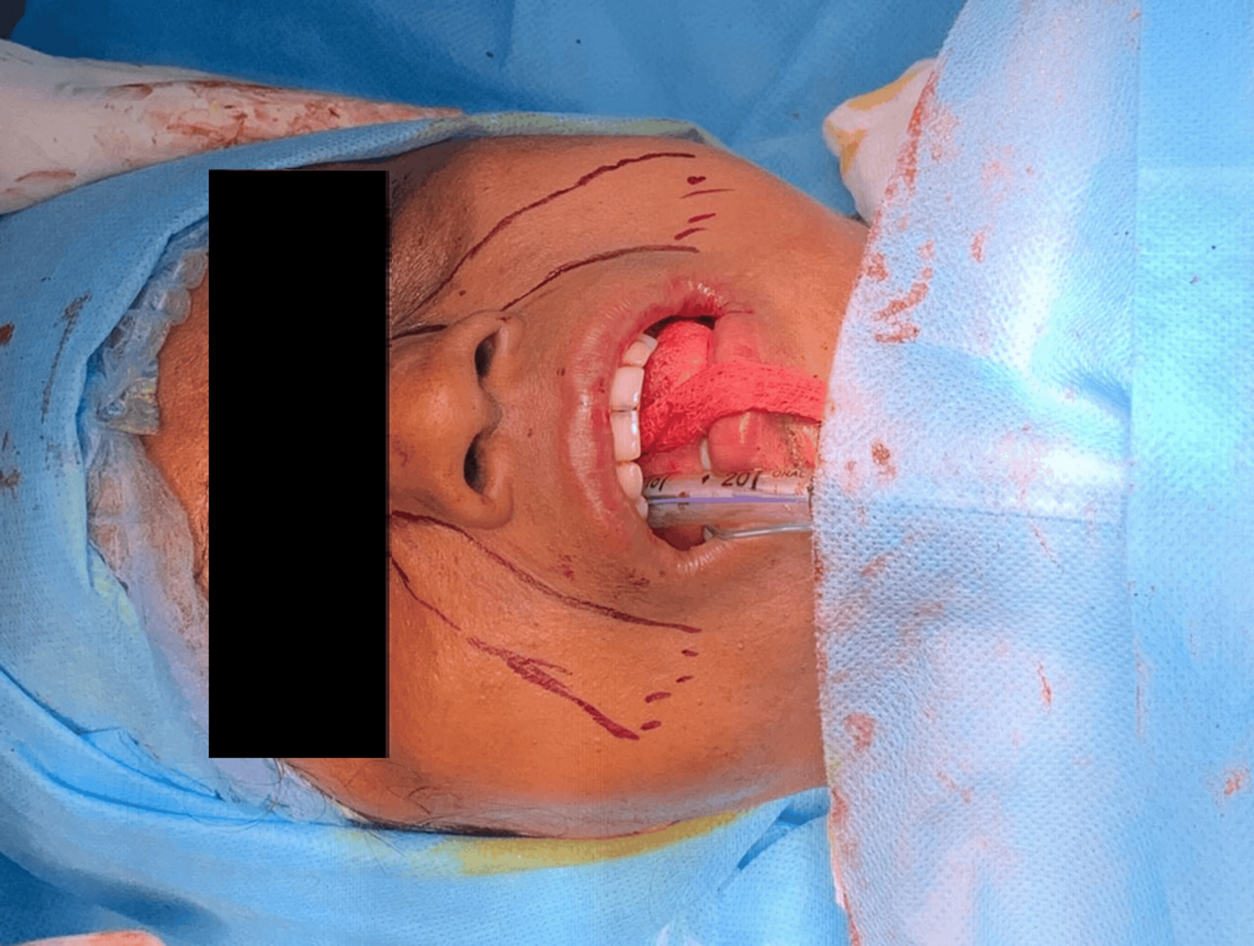
Marking for inferiorly based nasolabial flap harvest

**Figure 3:**
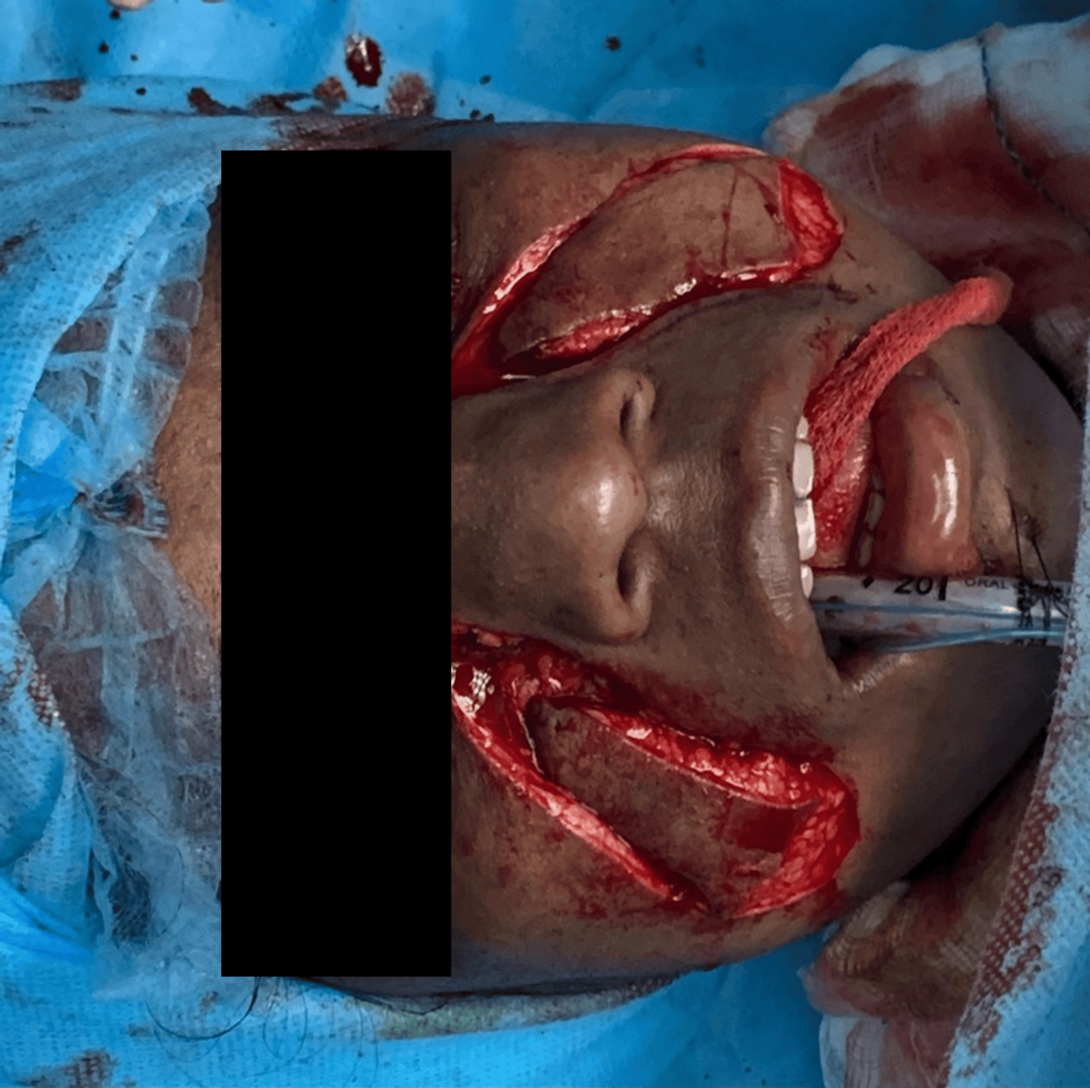
Nasolabial flap harvesting

**Figure 4:**
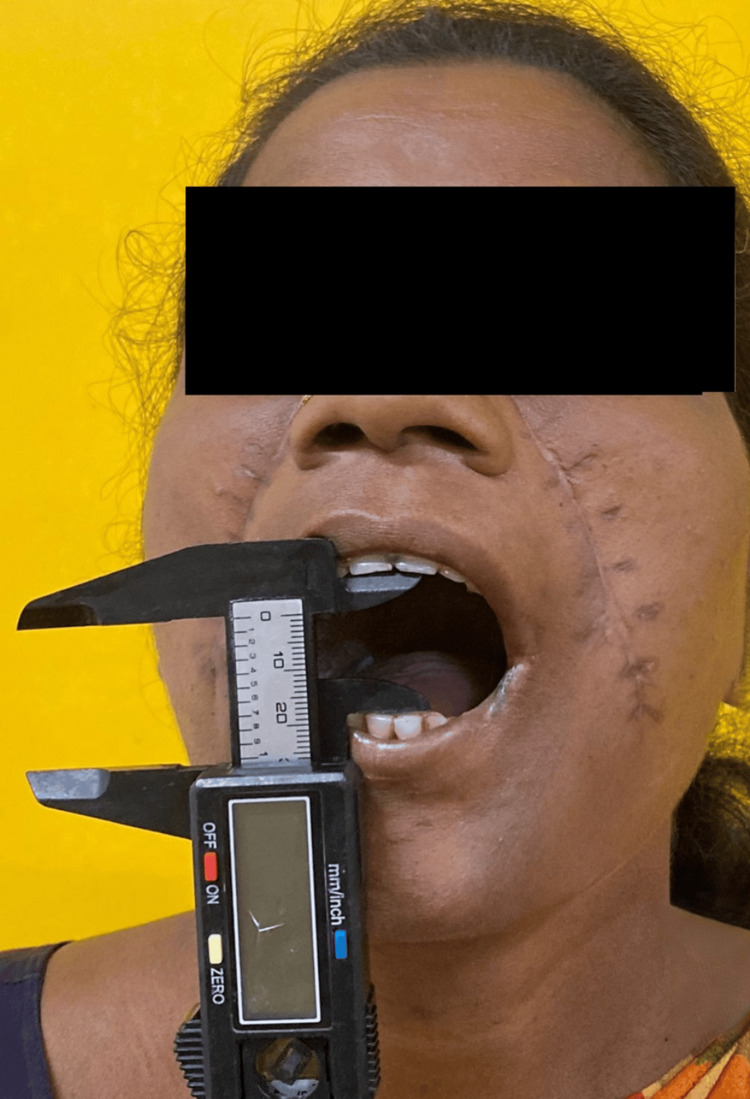
Postoperative mouth opening

The patient was then prepared for surgery; 2 mL of 0.2% ropivacaine infiltration was given subcutaneously over the cricothyroid membrane, followed by a cricothyrotomy. Oral intubation was successfully carried out, with the cricothyrotomy kept as a contingency. Intraorally, bilateral fibrous bands were excised using monopolar cautery, and the extraction of teeth 18, 28, and 48 was performed. Then, a bilateral coronoidectomy was performed. The defect was reconstructed bilaterally with inferiorly based nasolabial flaps (Figures 2-4). The patient was subsequently transferred to the postoperative ward, where recovery proceeded without complications.

## Discussion

LAs function by obstructing nerve conduction, resulting in a temporary loss of sensation in the targeted region. The underlying mechanism initiates with the displacement of calcium ions from nerve receptor sites, permitting the LA molecule to bind to these sites. This binding induces blockage of voltage-gated sodium channels (VGSCs), which prevents sodium ions from entering the nerve cell. Consequently, there is a reduction in sodium conductance and a depression in the rate of electrical depolarization. This means that the LA inhibits the nerve from attaining threshold potential, thus inhibiting the generation of an action potential, eventually leading to conduction blockade. This procedure effectively hampers the transmission of pain signals, culminating in localized anesthesia [7].

Failure of local anesthesia may stem from various factors, including variations in nerve anatomy, inflammation, infection, incorrect dosage or concentration, inadequate duration of action, improper injection technique, intravascular injection, and pseudocholinesterase deficiency, among others.

Local anesthesia resistance occurs when the patients do not obtain sufficient analgesia despite the appropriate administration of anesthetic agents. Mutations in VGSC genes such as SCN5A (Nav1.5) modify channel properties, thereby reducing the ability of LA to block nerve impulses. In EDS, where there is a defect in connective tissue, the diffusion and pharmacokinetics of LAs are hindered. Increased sensitivity to pain due to altered nerve responses contributes to reduced anesthetic efficacy in EDS patients. In addition to this, the presence of fragile capillaries and altered blood flow may influence the metabolism and clearance of anesthetic solution, further compromising the effectiveness. Vasodilation will hasten the diffusion of LA drugs away from the area, thus reducing their efficacy. People with EDS often have chronic, widespread mast-cell activation (with increased inflammation), which may explain why some LAs can work differently than others in people with EDS [8].

One another instance that displayed resistance to LA is the history of scorpion bites. The exact mechanism for the same is still elusive. Several hypotheses have been formulated based on the effects of scorpion venom on the nervous system. The scorpion venom has neurotoxins that target a variety of voltage-gated ion channels and induce changes in their binding properties, thus altering their functionality. Since LAs exhibit their effect through the binding and blocking of VGSCs, any structural and functional modifications to these channels have the ability to potentially impede the binding affinity of LAs, thereby reducing their effectiveness [4].

Scorpion venom delays the inactivation of the sodium channels, leading to persistent depolarization. As a result, the nerve remains in a hyperexcitable state. For an optimal binding, the ion channels have to be either in a closed or inactive state. This altered excitability hampers the stability of the neuronal membrane. Eventually, the neurons affected by scorpion venom show decreased response to LA, thus leading to its resistance. The scorpion venom triggers local and systemic immune responses, releasing proinflammatory cytokines and neuroactive substances that can modulate pain pathways. Recurrent exposure to scorpion venom induces changes in the local environment around the nerve, like an increase in inflammatory mediators or changes in tissue pH, altering the diffusion and penetration of anesthetic into nerve tissue, hence affecting its efficacy. Prolonged or repeated exposure to scorpion venom may result in plastic changes in nociceptive neurons, thus modifying pain signaling pathways in ways that could diminish the effectiveness of LAs. These changes may involve upregulating alternative ion channels or receptors that exhibit lower sensitivity to conventional LAs. The enhanced activity of compensatory sodium channel subtypes like Nav1.8 or Nav1.9, which are less sensitive to traditional LAs, can provide an alternative pathway for nerve depolarization, potentially leading to anesthetic resistance. In cases of chronic pain and nerve injury, upregulation of the sodium channels has been observed. Even though resistance to lignocaine is seen, they maintain excitability in sensory neurons even when other sodium channels are blocked [9,10].

Structural damage or remodeling of nerve fibers due to recurrent envenomation could alter nerve conduction properties. Changes in nerve fiber integrity may affect the distribution of LA molecules within the nerve sheath, resulting in an incomplete or patchy block. Since scorpion venom is known to induce a significant inflammatory response, the release of cytokines and infiltration of inflammatory response causing local tissue damage and chronic inflammation can lead to fibrotic changes in various tissues, including nerves. Fibrosis induced by scorpion venom or modifications in the extracellular matrix of nerve tissue may also impose a physical barrier to the diffusion of LAs, limiting their ability to reach and effectively block sodium channels [11].

Studies have shown that in people with a previous history of scorpion bites, bupivacaine alone or in combination with a drug like clonidine can be used to achieve regional anesthesia, or ropivacaine can be used [12,13]. Molecular modeling of ropivacaine has shown differences in the binding of the aromatic parts to VGSCs compared to another LA binding with VGSCs. The aromatic part of ropivacaine aligns toward the outer side of VGSCs, while the aromatic part of bupivacaine aligns toward the inner side of the channel. This differential alignment of the aromatic ring, along with its action on gamma-aminobutyric acid A and N-methyl-D-aspartate receptors, enables the LA action of ropivacaine, reducing the chances of resistance [13].

In this case, the patient originates from a place where scorpion bites are prevalent. Since the scorpion bite, the patient did not undergo any medical procedure under local anesthesia. Therefore, the patient did not know about the resistance to lignocaine. After lignocaine was found ineffective as an LA, other LAs like bupivacaine and ropivacaine were tried with patch test intradermally, and only ropivacaine was found successful as an LA. Not only can a repeated scorpion bite produce lignocaine resistance, but a single scorpion bite from a decade ago can also cause lignocaine resistance.

Due to the lack of availability and monetary issues, electrophysiological studies were not performed. This is a case report of a single case. The authors recommend more studies to be done on patients reporting a scorpion bite.

## Conclusions

To conclude, in addition to taking a medical history and previous dental history, we highly recommend that dental surgeons document the history of any previous scorpion bite. This history-taking should not be neglected to minimize the occurrence of multiple LA failures. Also, it should be routine practice to administer a test dose of appropriate LA in patients with a history of scorpion bites.
